# The barrier-protective effect of β-eudesmol against type 2-inflammatory cytokine-induced tight junction disassembly in airway epithelial cells

**DOI:** 10.1371/journal.pone.0302851

**Published:** 2024-04-30

**Authors:** Phuntila Tharabenjasin, Aekkacha Moonwiriyakit, Jenjira Sontikun, Kanokphorn Timpratueang, Suhaibee Kuno, Thitinan Aiebchun, Nathjanan Jongkon, Rungrawee Mongkolrob, Noel Pabalan, Kiattawee Choowongkomon, Chatchai Muanprasat

**Affiliations:** 1 Chulabhorn International College of Medicine, Thammasat University (Rangsit Campus), Klongluang, Pathumthani, Thailand; 2 Chakri Naruebodindra Medical Institute, Faculty of Medicine Ramathibodi Hospital, Mahidol University, Bang Phli, Samut Prakan, Thailand; 3 Department of Biochemistry, Faculty of Science, Kasetsart University, Bangkok, Thailand; 4 Department of Social and Applied Science, College of Industrial Technology, King Mongkut’s University of Technology North Bangkok, Bangkok, Thailand; Charotar Institute of Applied Sciences: P D Patel Institute of Applied Sciences, INDIA

## Abstract

Allergic inflammation, which is the pathogenesis of allergic rhinitis and asthma, is associated with disruption of the airway epithelial barrier due to the effects of type 2 inflammatory cytokines, i.e. interleukin-4 and interleukin-13 (IL-4/13). The anti-allergic inflammatory effect of β-eudesmol (BE) on the tight junction (TJ) of the airway epithelium has not previously been reported. Herein, the barrier protective effect of BE was determined by measurement of transepithelial electrical resistance and by paracellular permeability assay in an IL-4/13-treated 16HBE14o- monolayer. Pre-treatment of BE concentration- and time- dependently inhibited IL-4/13-induced TJ barrier disruption, with the most significant effect observed at 20 μM. Cytotoxicity analyses showed that BE, either alone or in combination with IL-4/13, had no effect on cell viability. Western blot and immunofluorescence analyses showed that BE inhibited IL-4/13-induced mislocalization of TJ components, including occludin and zonula occludens-1 (ZO-1), without affecting the expression of these two proteins. In addition, the mechanism of the TJ-protective effect of BE was mediated by inhibition of IL-4/13-induced STAT6 phosphorylation, in which BE might serve as an antagonist of cytokine receptors. *In silico* molecular docking analysis demonstrated that BE potentially interacted with the site I pocket of the type 2 IL-4 receptor, likely at Asn-126 and Tyr-127 amino acid residues. It can therefore be concluded that BE is able to prevent IL-4/13-induced TJ disassembly by interfering with cytokine-receptor interaction, leading to suppression of STAT6-induced mislocalization of occludin and ZO-1. BE is a promising candidate for a therapeutic intervention for inflammatory airway epithelial disorders driven by IL-4/13.

## Introduction

Allergic respiratory inflammation is a pathophysiological process in response to allergen exposure which leads to perturbation of the barrier function of airway epithelial cells [1, 2]. The breach of airway epithelial integrity by inflammation is postulated to contribute to several diseases, such as asthma and allergic rhinitis [3–6]. Dysregulated interaction between mucosal epithelia and the immune response, mediated by antigen-stimulated lymphocytes and various cytokines, plays a crucial role in allergic reactions [7, 8]. In particular, cytokines derived from T-helper lymphocytes (Th2-cell) and type 2 innate lymphoid cells, such as interleukin (IL)-4 and IL-13, are accepted to be major immunomodulators involved in promoting allergic inflammation and helminth infection [9–11]. It has been reported in the literature that these cytokines affect the airway epithelia by promoting goblet cell proliferation, activating inflammation, stimulating mucus hypersecretion, and disturbing tight junction properties [12, 13].

Tight junctions (TJ) play a crucial role in maintaining the integrity, polarity, and paracellular permeability of the airway epithelial lining. Disruption of TJs results in the entry of noxious substances into the airway submucosa [4, 5, 14, 15]. The key components of TJ are integral membrane proteins including occludin and claudins, as well as scaffold proteins including zonula occludens-1 (ZO-1), whose dynamic localizations primarily depend on actin cytoskeleton alignment [5, 16]. Accumulating evidence suggests that disruption of the TJ complex is involved in airway allergic inflammation. Previous studies have demonstrated that IL-4/13 interferes with the expression of TJ components, thereby compromising barrier function [17–20]. Occludin and ZO-1 expression have been found to be downregulated in mouse models of eosinophilic asthma [21]. Similarly, studies of bronchial epithelial cells isolated from asthmatic patients reported decreased expression of occludin and ZO-1 compared to cells from healthy subjects [18].

IL-4 and IL-13 are prototypical four-helix bundle short-chain cytokines which share functional signaling receptor chains [22–24]. IL-4 activates two types of receptors: type 1 IL-4 receptor (IL-4R), which is composed of heterodimeric complex between IL-4Rα and common γ -chain (γ_c_); and type 2 IL-4R, a heterodimer consisted of the IL-4Rα component paired with the IL-13 receptor α1-subunit (IL-13Rα1) [13, 22, 25]. IL-13 primarily executes its effects through the type 2 IL-4R, where IL-13Rα2 is responsible for a decoy receptor that binds specifically to IL-13. Of particular importance, there are three possible binding sites of type 2 IL-4R which recognize IL-4 and 13 [22, 26]. Site I is on the IL-4Rα subunit, whereas site IIa and site III are on the IL13Rα1 subunit. Site IIb is an area for the receptor-receptor interactions (IL-4Rα-IL-13Rα1) within the ternary complexes. Accordingly, interference in IL-4/13 receptor activation or its signal transduction may be beneficial in alleviating TJ dysregulation and preserving tight junction integrity [15, 22].

Traditional herbal medicines have been implemented in the treatment of various ailments due to their cost effectiveness, accessibility, and low risk of side effects [27]. β-eudesmol (BE), one of bioactive sesquiterpenoids isolated from the rhizome of *Atractylodes lancea* (Thunb.) DC. (A. lancea: Khod-Kha-Mao or Cang zhu) has been shown to exert anti-allergic, anti-inflammatory, and immunomodulatory activities [28–31]. BE suppresses inflammation induced by tumor necrosis factor alpha (TNF-α) and histamine in mast cells through regulation of the p38 MAPK pathway [28, 29]. Moreover, BE modulates macrophage lysosomal enzyme activity, nitric oxide release, and lymphocyte proliferation [30].

The role of BE in the anti-allergic inflammation of airway epithelial cells is currently unknown. The present study therefore aimed to investigate the preventive effect of BE against allergic inflammation-induced disruption of the TJ in a human bronchial epithelial cell line. Since protective outcomes of BE were observed in TJ integrity assessment and permeability studies, we also sought to determine the possible underlying mechanisms by which BE prevents TJ disassembly and performed *in silico* modelling to predict how BE interacts with proteins in cytokine signaling.

## Materials and methods

### Chemicals and reagents

BE was purchased from Sigma-Aldrich (St. Louis, MO, USA). Fetal bovine serum (FBS), trypsin, penicillin, and streptomycin were purchased from Life Technologies (Carlsbad, CA, USA). Minimum essential medium (MEM) was from Thermo Fisher Scientific Inc. (Waltham, MA, USA). Other chemicals were purchased from Tocris Bioscience (Bristol, UK), MedChemExpress (NJ, USA), Life Technologies (Carlsbad, CA, USA), and Sigma-Aldrich (St. Louis, MO, USA).

### Cell line and cell culture

The 16HBE14o- cell line was from Merck Millipore (Darmstadt, Germany) and cultured in MEM. The medium was supplemented with 1% non-essential amino acid, 10% FBS, 100 U/mL penicillin, and 100 mg/mL streptomycin (Life Technologies, Carlsbad, CA, USA). The cells were propagated in a 75-cm^2^ T flask (Corning, NY, USA) in a humidified atmosphere containing 95% O_2_ and 5% CO_2_ at 37˚C. The cells with a passage number of 25 to 35 were used for all experiments.

### Measurement of tight junction integrity

Transepithelial electrical resistance (TEER) indicates the integrity of epithelial tight junctions. Confluent monolayers of 16HBE14o- cells at initial density of 5.0 × 10^5^ cells/well were grown on a polyester 0.4-μm pore size membrane of 12-mm diameter Transwell inserts (Corning Life Sciences, Tewksbury, MA, USA). Each insert was placed in a well in a 24-well plate Transwell chambers (Corning, NY, USA) with 250 μl of medium in the apical (upper) chamber and 750 μl medium in the basolateral (lower) chamber. The culture media was freshly replaced daily for 14 days until the TEER of the monolayers was ≥ 900 Ω.cm^2^, indicating the complete formation of epithelial TJs.

TEER was measured at baseline before treatment. Thereafter, the medium was replaced with FBS–free medium in the control group. In the treatment group, the cells were pre-incubated for 1 h with vehicle (0.25% v/v dimethyl sulfoxide; DMSO) or BE prior to exposure to a mixture of IL-4 and IL-13 (IL-4/13) (10 ng/mL each). TEER was then measured at 0, 24 h, and 48 h of incubation. Serially diluted BE in FBS–free medium was added either to the apical or basolateral compartment of the wells at final concentrations of 5 μM, 10 μM, and 20 μM. TEER was measured by EVOM2 volt-ohm meter (World Precision Instruments, Sarasota, Florida, USA).

### Assessment of epithelial paracellular permeability

Epithelial permeability across polarized 16HBE14o- monolayers was assessed by measuring the flux of fluorescein isothiocyanate- (FITC)-label dextran (molecular weight 4 kDa, Sigma-Aldrich) and Texas-red-dextran (molecular weight 10 kDa, Sigma-Aldrich) from the apical to basolateral compartments. At 48 h after treatment, the upper and lower compartments of treated media were aspirated. Thereafter, serum-free media with either 1 mg/mL FITC- or 50 μg/mL Texas-red-conjugated dextran was directly added into the apical compartment. Paracellular flux was assessed by taking 100 μL aliquots from the basolateral compartment after 2 hours of sterile incubation at 37°C. The amount of fluorophore that diffused into the lower chamber was measured by the fluorescent microplate reader (Synergy™ Neo2 multimode plate reader, BioTek Instruments) at an excitation and emission wavelength of 485 nm and 535 nm, respectively. The FITC- and Texas-red-conjugated dextran concentration was calculated with a known standard concentration curve.

### Cell viability assays

The viability of 16HBE14o- cells treated with various concentrations of BE was assessed using a 3-(4,5-dimethylthiazol-2-yl)-2,5-diphenyltetrazolium bromide (MTT) colorimetric assay. In brief, the cells were grown overnight on 96-well plates at a seeding density of 5 x 10^5^ cells/well. Cell viability was then determined after replacement of the media with serum–free media as a control, or serum–free media containing IL-4/13 (10 ng/mL each) plus vehicle or BE at concentrations ranging from 10–20 μM for a further 48 h.

To assess cell viability, the MTT solution (catalog no. M5655; Sigma) was added to obtain a final concentration of 0.5 mg/mL and incubated for 2 h to generate formazan crystals, which were dissolved with dimethyl sulfoxide. Colorimetry of blue formazan was detected by measuring absorbance at 540 nm using a microplate reader (Victor 2V, PerkinElmer, Waltham, MA, USA). The fluorescent signal was directly proportional to the percentage of cell viability, which was calculated by comparing the absorbance of the treated group to the control group.

### Western blot analysis

The 16HBE14o- cell monolayers were grown in cell culture plates at a density of 5 × 10^5^ cells/mL for 14 days. Protein extraction and immunoblotting protocols were performed as previously described [32]. After 48-h exposure to each treatment, cells were washed with cold 1xPBS and lysed on ice in radioimmunoprecipitation assay buffer with protease and phosphatase inhibitor cocktails, followed by scraping with a rubber spatula. The cells were transferred to a microcentrifuge tube and the lysate clarified by spinning for 20 minutes at 12,000 revolutions per minute at 4°C. Sodium dodecyl sulfate polyacrylamide gel electrophoresis (SDS-PAGE) (Invitrogen) was used to separate 25 μg of protein samples before transferring them to a nitrocellulose blotting membrane (GE Healthcare, Texas, USA). After being blocked at 25°C for 1 h with 5% non-fat milk (Bio–Rad, Hercules, CA, USA) in Tris-Buffered Saline and 0.1% (vol/vol) Tween-20 (TBST), the membranes were probed overnight at 4°C with occludin (1:1000, catalog number ab31721), ZO-1 (1:1000, catalog number 33–9100), STAT6 (1:1000, catalog number 9362S), pSTAT6 (1:1000, catalog number 56554S), and β-actin (1:1000, catalog number 4970S) (Cell Signaling, Boston, MA, USA). After washing with TBST four times, the membranes were incubated with 2.5% non–fat dried milk plus appropriate horseradish peroxidase-conjugated anti-rabbit IgG (1:20000, catalog number ab6721, Abcam, Cambridge, MA,) or anti-mouse (1:3000, catalog number 7076S, Cell Signaling, Boston, MA, USA) secondary antibodies for 1 h at 25°C. Proteins on the immunoblots were visualized with an enhanced chemiluminescent substrate reagent according to the manufacturer’s instructions (Merck Millipore, Billerica, MA, USA). The signal was captured by using ChemiDoc (Bio-Rad Laboratories, Hercules, CA, USA) to determine the densitometric ratio, and beta-actin expression was used as a loading control.

### Immunofluorescent imaging

Immunofluorescent staining was performed at 48 h post-treatment. Briefly, cells were washed with phosphate−buffered saline (PBS), and fixed in 4% paraformaldehyde for 10 min.

The permeabilizing agent used was 0.1% Triton-X 100, for 10 min. The cells were blocked in PBS with 5% fetal bovine serum for 30 min at room temperature, then incubated at 4°C overnight with a mixture of 1:200 occludin antibodies (Invitrogen, catalog number 40–4700) and 1:200 anti–ZO–1 antibodies (Invitrogen, California, USA; catalog number 339100). The cells were subsequently incubated with fluorophore-conjugated secondary antibody (Ab) (1:1000) (Invitrogen, catalog number A-11008 and A32727) at room temperature for 1 hr. The cells were counter-stained with Hoechst for nuclei. Images were captured using Confocal-integrated Opera Phenix Plus High-Content Screening System (PerkinElmer). The degree of co-localization, based on the presence of ZO–1 spots in the occludin area and vice-versa, were determined from nine visual fields per experimental replication under the Opera Phenix software.

The high magnification confocal images (63X) were captured using an immunofluorescent microscope (ZEISS LSM 900 with Airyscan 2) in 60 z-stacks with an interval of 0.09 μm per stack. The projection was processed by Zen software version 3.8.

### Preparation of compound and receptors for *in silico* analysis of type 2 IL-4R and BE binding

The crystal structure of the cytokine IL-13 in a ternary complex with the receptors (IL-4Rα-IL-13Rα) was obtained from the Protein Data Bank (PDB) entry 3bpo with a resolution of 3.0 Å [26]. Missing atoms and residues in the structure were observed and rebuilt on the Swiss PDB Viewer [33]. The crystallographic waters and cofactors were removed from the structure using the Discovery Studio (DS) Visualizer v21.1.0.20298 (BIOVIA Dassault Systems, San Diego, USA). The cytokine (chain A) was deleted and only the cytokine receptors (Chain B and Chain C) were used in the docking simulation. The polar hydrogen atoms were added to the receptors with Kollman charges and converted to be pdbqt using AutoDockTools (ADT tools) software [34, 35]. The tools were also used to prepare a grid parameter file (GPF) of the proteins and a docking parameter file (DPF) of the ligand for the AutoDock calculation. In the GPF file, the center of receptors was used as the center of the grid box with the X, Y, and Z coordinates of −24.319, −12.904, and −36.611, respectively. The numbers of grid points in the box were set to be maximized by 126×126×126 and the spacing used was the default (0.375 Å). The ligand BE was retrieved from the NCBI database (www.ncbi.nlm.nih.gov), PubChem CID number 91457, in sdf format [36]. The ligand was cleaned and converted to pdb format via DS studio and subsequently changed into the pdbqt format by ADT tools prior to the docking study.

To perform blind docking and to find a possible binding site of BE, we duplicated the docking calculation using the whole receptor as its search space. The Larmarckian genetic algorithm (LGA) with the number of GA runs was 100 and 200. The initial coordinates and orientation of the ligand were random. The glg and dlg were done by autodock 4.2.6 in Linux system [34]. The interactions between the BE and receptors were visualized via Pymol [37], Chimera version 1.16 [38], and DS studio [39].

### Statistical analysis

Data are expressed as mean ± standard error of mean (SEM). Multiple comparisons were performed by one-way analysis of variance (ANOVA) followed by Dunnett’s post-hoc test. A two-factor repeated measure of ANOVA (two-way ANOVA) was used to compare the difference in TEER over time in each treatment. All data analyses were performed using GraphPad Prism 9.0 (GraphPad Software Inc., San Diego, CA, USA). The level of significance for all statistical tests was set at *P*-value < 0.05 (two-tailed).

## Results

### BE prevents IL-4/13-induced epithelial barrier disruption

**Fig 1:**
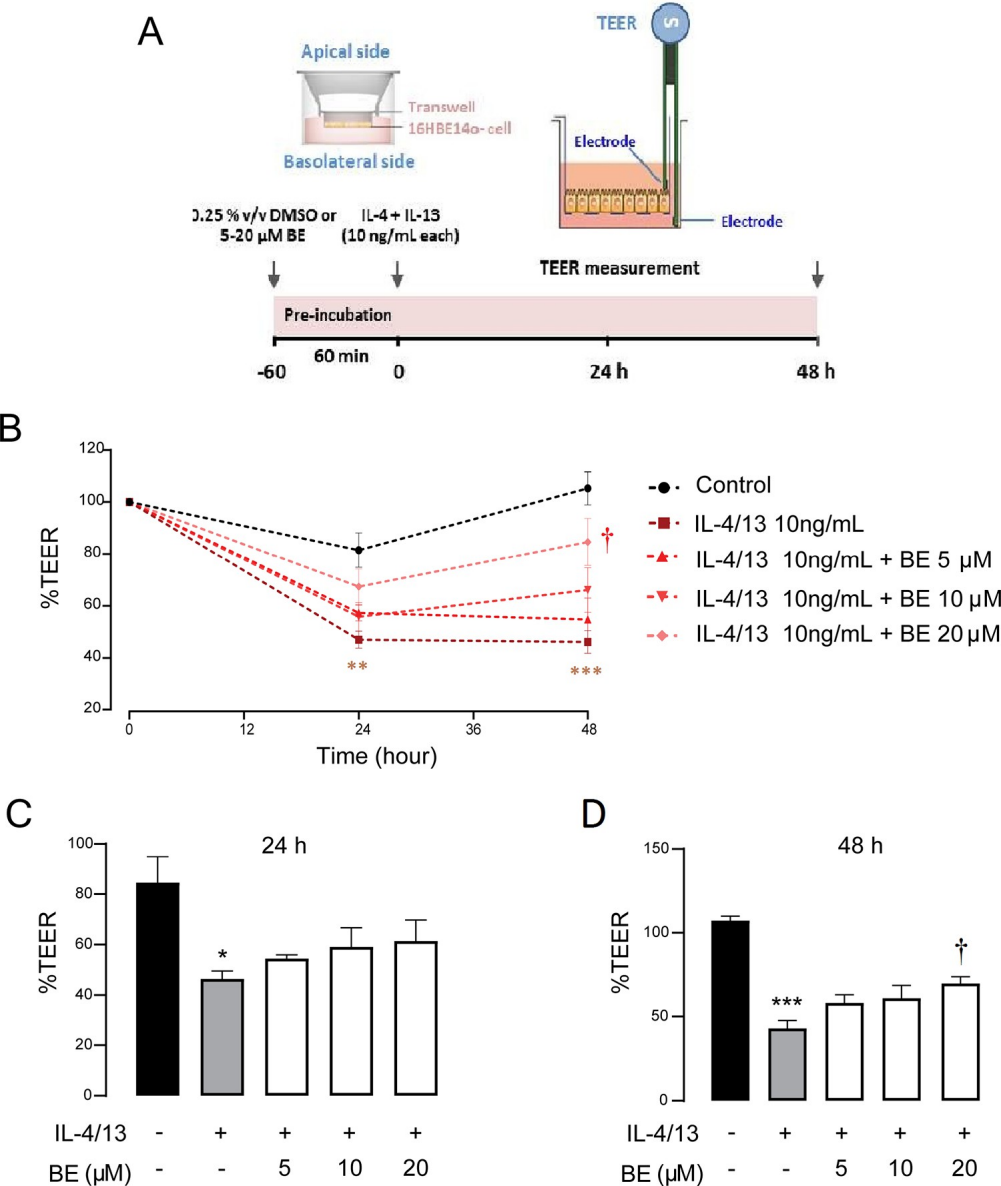
The preventive effect of BE on IL-4/13-induced epithelial barrier disruption in 16HBE14o- cells. (A) Schematic diagram of barrier integrity measurement. The TEER of the indicated treatment was obtained from pretreatment of cells with vehicle (0.25% v/v DMSO) or BE at dose of 5, 10, and 20 μM prior to exposure to IL-4/13 (10 ng/ml each). (B) Sequential change of TEER after each treatment. (C–D) Observations for the TEER after 24 h and 48 h, respectively. The data represents the mean percentage of changed TEER to baseline ± S.E.M (n = 5–8). Differences in mean values over time were analyzed using two-way ANOVA and Tukey’s post-hoc analysis in Fig 1B, and one-way ANOVA with Dunnett’s post-hoc analysis in Fig 1C and 1D. * *P* < 0.05,** *P* < 0.01, *** *P* < 0.001 compared with control group (black bar); ^†^ *P* < 0.05 compared with vehicle-pretreated group (gray bar).

To determine the protective effect of BE on cytokine-induced barrier disruption, change in epithelial integrity was evaluated by TEER measurement. As shown in Fig 1, the sequential TEER of 16HBE14o- monolayers in the untreated group remained unchanged, while the level of TEER was markedly reduced after exposure to IL-4/13 (10 ng/mL each) for 24 h (*P* < 0.01) and 48 h (*P* < 0.001), indicating an induction of barrier disruption (Fig 1C and 1D). Interestingly, pre-treatment with 5 μM– 20 μM of BE increased the TEER in concentration- and time- dependent manners, and 20 μM of BE significantly inhibited the effect of the cytokines. These results suggest that BE could prevent IL-4/13-induced barrier disruption by exerting the most significant effect at the duration of 48 h and a concentration of 20 μM.

### Effect of BE on 16HBE14o- bronchial epithelial TJs permeability

Since TJ plays key role in regulating paracellular permeability and maintaining barrier integrity, the barrier-protecting effect of BE could be mediated through resolving TJ. We therefore assessed the permeability of airway epithelial monolayers by determining the flux of the administered macromolecules, FITC-conjugated dextran (4 kDa) and Texas-red- conjugated dextran (10 kDa), from the apical to basolateral compartment over time.

**Fig 2:**
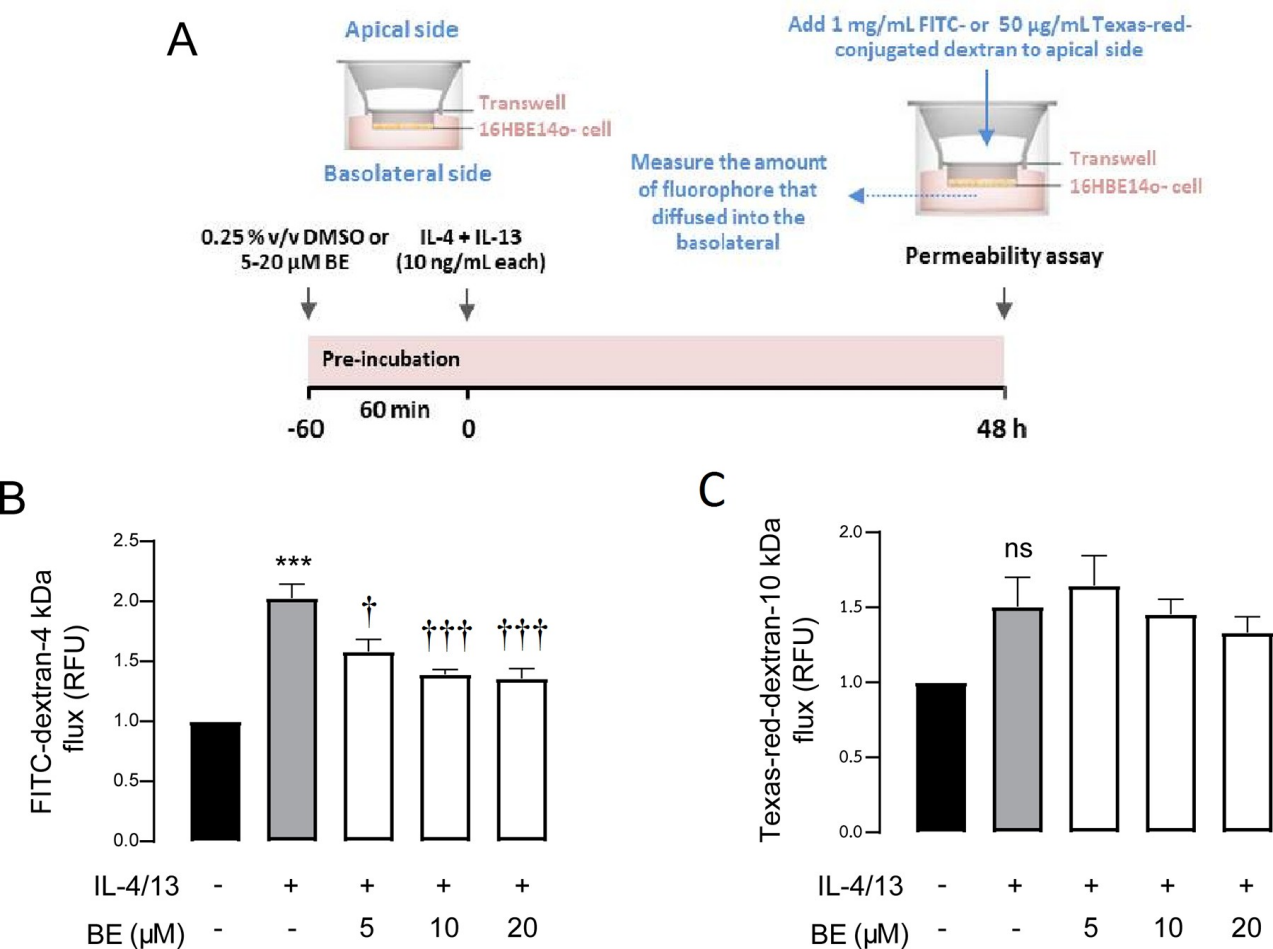
The effect of BE on barrier integrity and tight junction assembly determined by permeability assay. (A) Schematic diagram of the permeability assay. After 48 h exposure to the indicated treatment, 4-kDa-FITC-dextran (B) or 10-kDa-Texas-red-dextran (C) was added to the apical chamber. The fluorescence intensity of FITC- or Texas-red conjugated dextran leaking from the upper to the lower chambers of the Transwell membranes was measured by sampling media from the basolateral chamber at 2 h. Data are expressed as means of relative fluorescent unit (RFU) of control ± S.E.M (n = 5–6). *** *P* < 0.001 compared with control group (black bar); ^†^ *P* < 0.05 and ^†††^ *P* < 0.001 compared with the vehicle-pretreated group (gray bar) (one-way ANOVA with Bonferroni multiple comparison test).

As depicted in Fig 2, in the absence of both BE and IL-4/13 after 48 h of incubation, the flux of FITC- and Texas-red- conjugated dextran was unchanged, indicating an intact barrier. IL-4/13 significantly (*P* < 0.001) increased FITC- (2.00 ± 0.02 RFU), but not Texas-red- (1.49 ± 0.03 RFU) flux. Pre-exposure to BE significantly diminished (*P* < 0.05–0.001) the FITC- fluxes induced by IL-4/13. The FITC- fluxes were 1.62 ± 0.02, 1.49 ± 0.08, and 1.48 ± 0.07 RFU after BE treatments at 5, 10, and 20 μM, respectively. However, Texas-red- flux was unchanged. Of note, the alteration in flux of 4 kDa dextran, but not 10 kDa would suggest that the impaired barrier function could be a result of TJ disassembly rather than cell injury or death. These results strongly supported the result of TEER experiment showing, an alleviative effect of BE against IL-4/13-induced TJ disruption.

### Cytotoxicity evaluation of BE and in combination with IL-4/13 treatment

**Fig 3:**
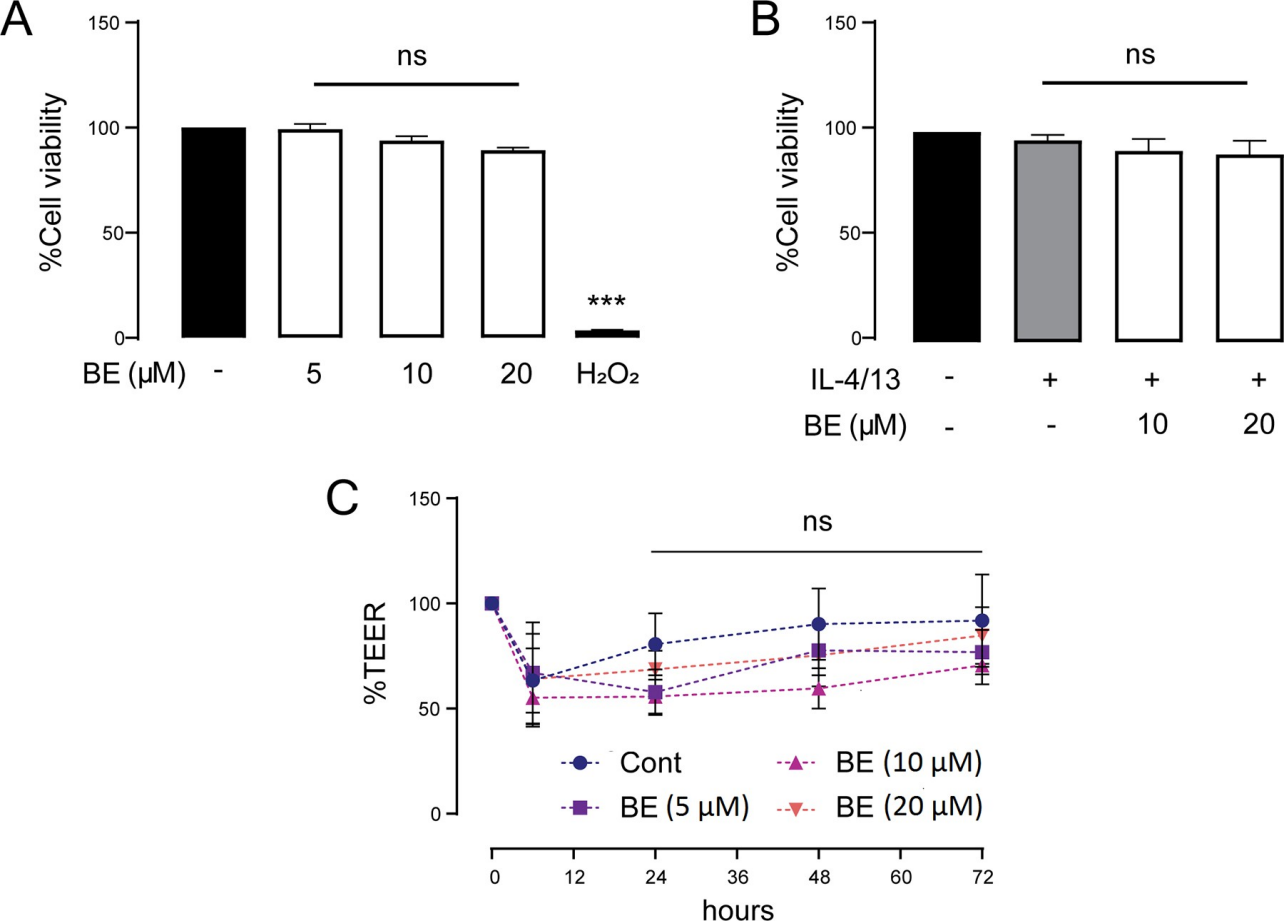
Combining effects of BE and IL-4/13 at different concentrations on 16HBE14o- cell survival. Cell viability was determined by MTT assay after treatment with BE of various concentrations (5–20 μM) alone (A) or in combination with IL-4/13 (each at 10 ng/mL) (B) for 48 h. (C) TEER was measured over time after a prolonged 72 h exposure to BE. The results are expressed as mean ± SEM (n = 4). *** *P* < 0.001 compared with control group (black bar) (one-way ANOVA); ns: non-significant *P*-value.

The increased epithelial permeability and decreased TEER caused by cytokines may be consequences of either TJ disruption or cell injury. It is important to exclude the possibility that both BE and IL-4/13 affect cell viability. To investigate whether the anti-inflammatory effect of BE involved prevention of cytotoxicity, MTT assays were performed by treating cells with varying concentrations of BE (0–20 μM) or co-treatment with IL-4/13 (10 ng/mL each) for 48 h. The medium containing 0.05 mM hydrogen peroxide (H_2_O_2_) served as a positive control. As shown in Fig 3A and 3B, the percentage of viable cells in all individual treatments was not different from the control, suggesting that neither the BE, IL-4/13, or the combination of both had any effect on cell viability. These results further supported that the inhibitory effect of BE on cytokine-induced TJ disruption did not involve altered cell survival. Moreover, we ascertained the physiological effect of BE on the airway epithelial barrier. As shown in Fig 3C, there was no significant difference in TEER after 72-h incubation at each concentration (5–20 μM) of BE, indicating that BE might not has any basal barrier protective effect.

Collectively, these results suggest that BE or IL-4/13 alone, or in combination, had virtually no cytotoxic effect on human bronchial epithelial 16HBE14o- cells.

### Effect of BE on TJ-associated protein expression

Previous studies have shown that both IL-4 and IL-13 impaired expression of tight junction protein components, including occludin and ZO-1, involved in regulating the permeability of the airways [17–20]. To explore the underlying barrier-protective mechanism by which BE impedes the barrier dysfunction induced by cytokines, we further examined the expression of TJ-associated proteins after exposure to IL-4/13 with or without pre-treatment with BE. In light of previous experiments, we opted to use 20 μM BE with an incubation period of 48 h.

**Fig 4:**
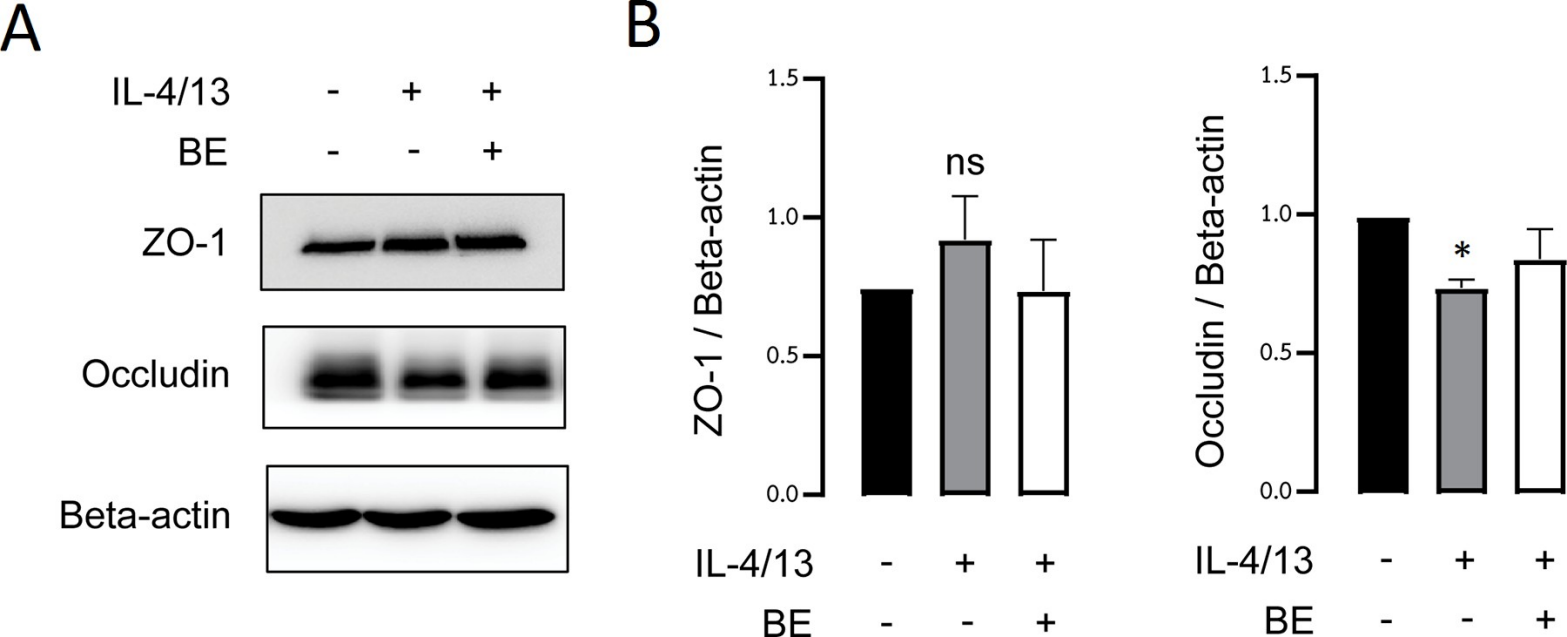
Barrier protective effects of BE might not be via alteration of TJ protein expression in 16HBE14o- cells. (A) Representative electrophoretic bands of the TJ proteins, ZO-1, and occludin (B) The densitometry analyses of TJ proteins in cells treated for 48 h with IL-4/13 (10 ng/mL each) alone or pre-exposure to 20 μM BE was analyzed by western blot analysis. The histogram bar represents the mean of the ratio of the control ± SEM to the three independent experiments. The values were normalized to beta-actin. * *P* < 0.05 compared with the control group (black bar) (one-way ANOVA); ns: non-significant *P*-value.

As shown in Fig 4 and data in S1 File, western blot analysis of total cell lysate showed that treatment with IL-4/13 alone resulted in notably significant decreased expression of occludin (*P* < 0.05). Although 20 μM of BE exhibited a trend of preserving IL-4/13-induced changes in expression, the difference was not statistically significant when compared to the control. Meanwhile, IL-4/13-treated cells with or without pre-incubation with BE had no difference in ZO-1 protein level. These results suggested that the protective effect of BE against TJ disruption induced by cytokines is unlikely to be mediated by alteration in TJ protein expression.

### Prevention of mislocalization of occludin and ZO-1 by BE treatment

To gain additional insights into the mechanisms underlying the preventive effect of BE on IL-4/13-induced barrier disruption, we further determined the TJ assembly by performing immunofluorescence to investigate the localizations of occludin and ZO-1.

**Fig 5:**
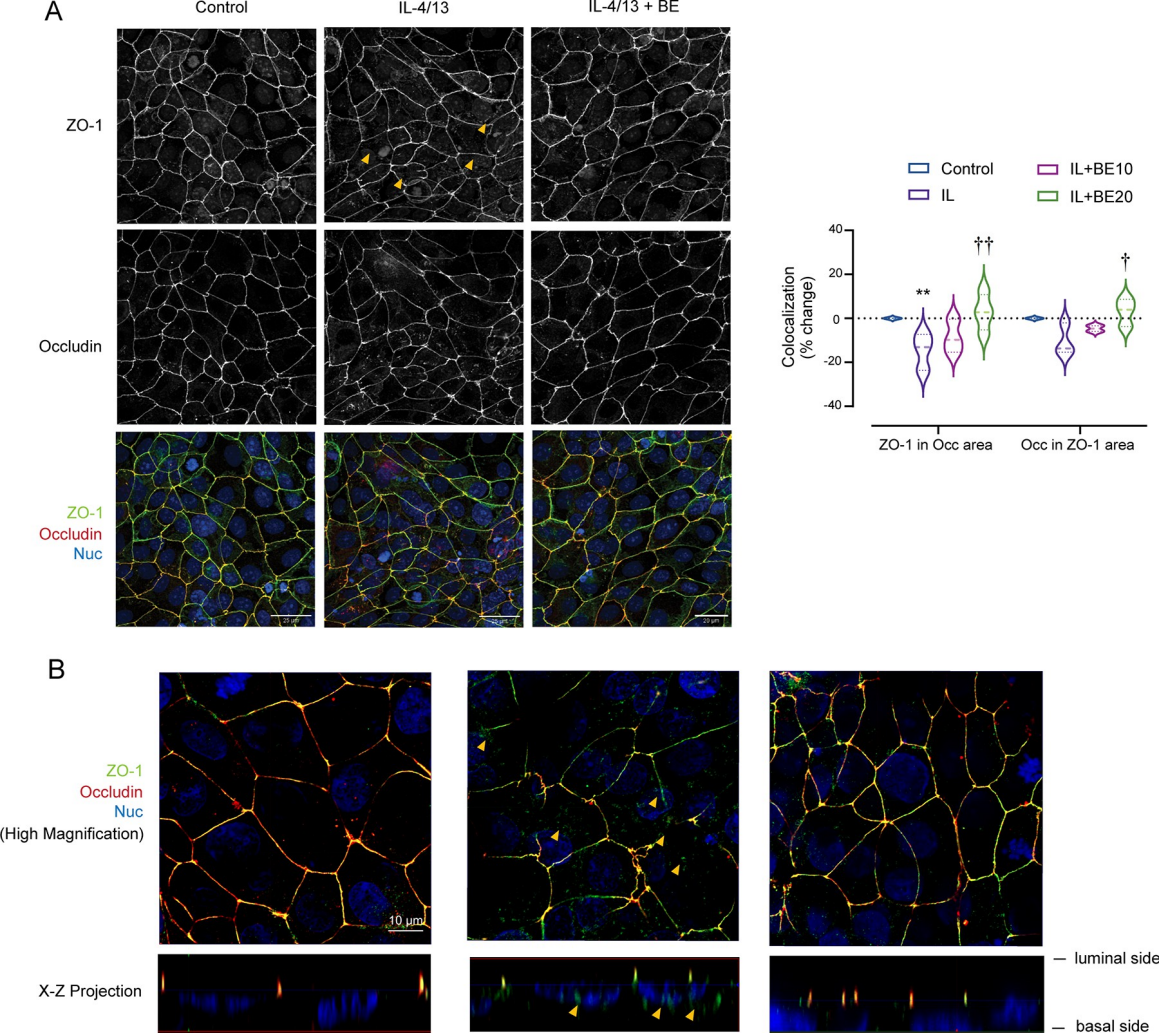
Immunofluorescence staining of TJ proteins. (A) Localization of occludin and ZO-1 labeled by immunofluorescence in monolayers treated with IL-4/13 (10 ng/mL each) (Middle panel) or the cytokine plus 20 μM BE (Right panel). Scale bars represent 25 μm. The regions of occludin and ZO-1 were marked and segmented, and intensities of ZO-1 that localized in the occludin region, as well as the occludin in the ZO-1 region, were calculated. The violin plot shows the mean percentage of change in the aforementioned intensities (Right panel). (B) The high magnification of tight junction images in X-Y and X-Z projection (scale bars represent 10 μm). The yellow arrowhead represents internalization of ZO-1 into cytoplasmic sites at 48 h after IL-4/13 treatment. ** *P* < 0.01 compared with control group; ^†^ *P* < 0.05, ^††^ *P* < 0.01 compared with vehicle-pretreated group (one-way ANOVA).

As depicted in Fig 5A and 5B, the control 16HBE14o- cells exhibited predominant colocalization of occludin and ZO-1 at the paracellular areas, indicating an intact apical junctional complex. Conversely, the colocalization of occludin and ZO-1 protein appeared to be perturbed in IL-4/13-treated cell at 48 h, which was manifested by reduced ZO-1 presence in the apical tight junction area. The high magnification projections indicated that ZO-1 appeared in more cytoplasmic regions (yellow arrow heads) (Fig 5B). Interestingly, pre-treatment with BE dose-dependently prevented mislocalization of these two proteins, in which fluorescence signals of occludin and ZO-1 remain in co-existence at the paracellular areas.

Taken together, these results indicate that BE preserves airway epithelial integrity by counteracting the effect of cytokines in inducing TJ disassembly.

### Involvement of STAT-6 inhibition as a mechanism of TJ preservation by BE

Upon cytokine activation, the Janus kinases (JAK)-signal transducer and activator of transcription (STAT) pathway is a major signal transduction pathway and STAT6 phosphorylation as the results of JAK1, JAK2, JAK3 and tyrosine kinase (TYK)2 is a well-known signaling mediator of IL-4 and IL-13 receptors in allergic inflammation [25, 40].

**Fig 6:**
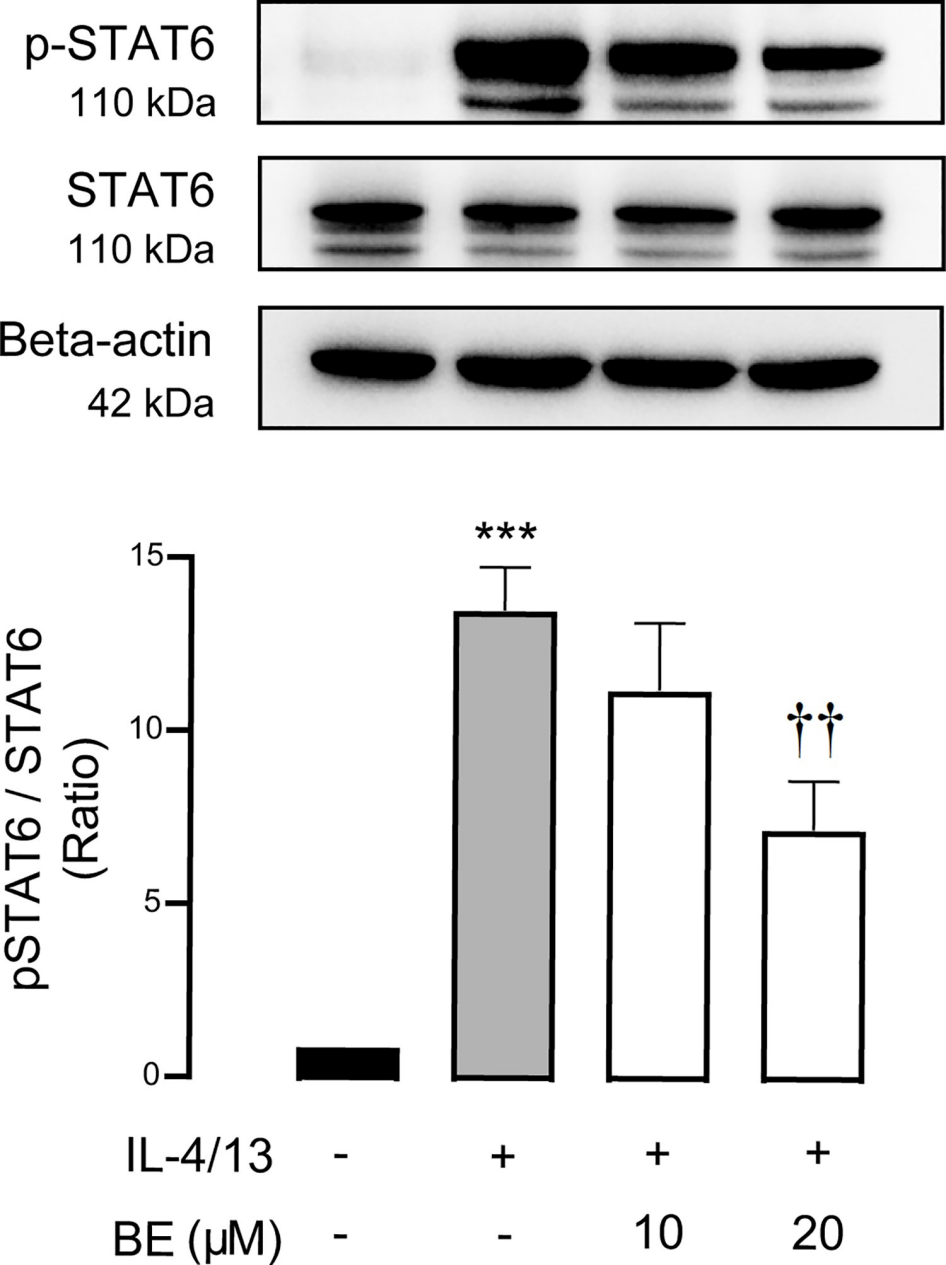
BE suppresses TJ disruption induced by cytokines through the inhibition of STAT6 phosphorylation. The western blot analyses of STAT6 and the phosphorylated form of STAT6 (pSTAT6) protein were derived from cell extracts after 48 h of the indicated treatment. Data is represented as the mean of the ratio of the control ± SEM of the three independent experiments. Values were normalized to STAT6. *** *P* < 0.001 compared with control group (black bar); ^††^ *P* < 0.01 compared with vehicle-pretreated group (gray bar) (one-way ANOVA).

As we found that BE counteracted the effect of cytokine-induced perturbation of barrier function, we hypothesized that BE would interrupt cytokine signaling pathways. To prove this speculation, we further evaluated phosphorylation of STAT6 (pSTAT6) levels, which indicate its activation, by western blot analyses. As shown in Fig 6 and data in S1 File, it was apparent that while the pSTAT6 protein band was not detected in control, the level of pSTAT6 was significantly increased (*P* < 0.001) after cytokine stimulation. The increased the ratio of pSTAT6 and STAT6 was significantly (*P* < 0.01) attenuated by pre-incubation with BE in a concentration-dependent manner. Collectively, these results indicate that the protective effect of BE on barrier disruption induced by IL-4/13 is via inhibition of STAT6, which may suggest antagonistic action of BE on cytokine receptors.

### *In silico* analysis of BE as anti-inflammatory compound targeting type 2 IL-4R

As our results demonstrated that BE interfered with IL-4/13 signaling, we performed *in silico* analysis to predict the interactive site of BE to type 2 IL-4 receptor complexes, which are formed by the heterodimer of IL-4R and IL-13Rα1.

**Fig 7:**
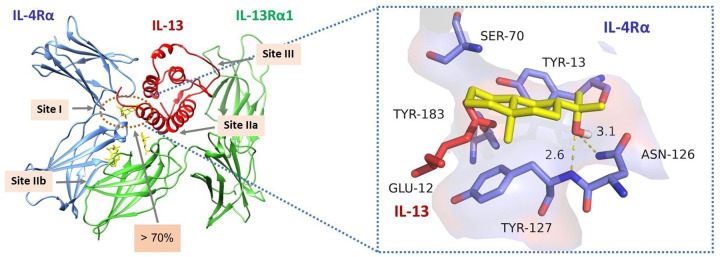
Representation of molecular docking between BE and site I binding pocket of type 2 IL-4R. (Left) 3D visualization of the four active pockets of the IL-4R; (Right) 3D visualization of BE (yellow structure) in site I pocket of IL-4R. BE was docked to the Asn-126 and Tyr-127 in > 70% of 200 runs and the orientation of BE at the site I pocket, near the Glu-12 of IL-13 (some residues were omitted for clarity).

Virtual screening using AutoDock was performed to identify the possible binding sites and calculate the binding affinity between the receptors and BE. The molecular docking results showed that BE was gathered at site I of IL-4Rα in 78.5% of 200 runs (72% of 100 runs) and with a binding energy of −5.86 kcal/mol for both calculations, as visualized in Fig 7. The site I binding site is composed of Tyr-13, Ser-70, Glu-72, and Tyr-183 interacting with Glu-12 and Arg-65 of IL-13 (or Glu-9 and Arg-88 of IL-4). We proposed that the physicochemical mechanism of BE in inhibiting IL-4/13 signaling pathway was indirectly binding to the site I pocket of IL-4R, probably at Asn-126 and Tyr-127, and the orientation of the ligand was near Glu-12 of IL-13.

## Discussion

Perturbation of the barrier function of the pulmonary epithelium mediated by Th2 cytokines, particularly IL-4/13, is considered to be a hall-mark characteristic in allergic inflammatory lung diseases such as asthma [15, 17–20, 41]. Thus far, the anti-allergic inflammatory properties of BE in the airway epithelium have never been addressed. We have provided empirical evidence, for the first time, that BE exerts a protective effect on IL-4/13-induced TJ disruption in the human airway epithelial 16HBE14o- monolayer. The underlying mechanism is primarily due to antagonistic action of BE to the IL-4/13 receptor, which in turn interrupts the cytokine downstream STAT6, thereby preventing mislocalization of the TJ proteins occludin and ZO-1. The *In silico* docking results showed dual blockage of IL4 and IL-13 with BE selectively at the binding site I of the type 2 IL-4 receptor, which consists of a heterodimer between IL-4Rα and IL-13Rα1 subunits [13, 22, 25].

Intact junctional complexes are relatively impermeable to ion flux and exhibits high electrical resistance [3, 42]. Our study demonstrated that IL-4/13 could induce barrier disruption, as shown by a reduction of TEER over time. These observations are consistent with the previous studies [17, 18, 20]. For example, Saatian and colleagues (2013) applied IL-4 to both the apical and basolateral compartments of 16HBE14o- cells. They found that the disruptive effect of IL-4 was dose-dependent, with the concentration ranging from 5–50 ng/mL over a 48–72 h incubation period. Moreover, IL-4/13 was shown to break down the TJ barrier of asthmatic primary human bronchial epithelial cells [18, 43], primary sinonasal epithelial layers [17], and human nasal and tracheal epithelial cells [6, 44].

As for assessing the permeability of TJ, macromolecular flux assays using 4 kDa FITC-conjugated dextran and 10 kDa Texas-red- conjugated dextran permeability is another parameter used to assess the impairment of TJ [42]. We could differentiate the magnitude of barrier disruption by difference in the molecular weight of fluorophore molecules. In principle, the molecular weight of a molecule is inversely proportional to its rate of diffusion [45]. The diffusion rate of a larger molecule with a higher molecular weight would be slower than that of smaller molecule. Flux of large molecule might suggest passage through an unrestricted pathway, which is caused by cell death. The finding that IL-4/13 significantly increased FITC-dextran flux, but not Texas-red- flux, in the vehicle-pretreated group could be supported by this concept and it could be postulated that altered monolayer integrity involves a change in TJ opening, rather than cellular damage. This hypothesis was also supported by the finding of cell viability experiment that cytokines did not exert the cytotoxic effect (Fig 3). It was therefore suggested that the observed alteration in the barrier integrity would rather be associated with the cell-cell apical TJ dynamic.

Several mechanisms may be responsible for the IL-4/13-induced disruption of epithelial barriers. They are not only involved in interfering with the transcriptional control of TJ proteins, but also with the intracellular localization of TJ proteins, even where the expressional level remains unaffected [6, 17, 20]. Our western blot analysis provides evidence that IL-4/13 significantly decreased the expression of occludin, and the decreasing levels were positively correlated with decreased integrity of the epithelial barrier as determined by TEER (Figs 1 and 4). This finding was inconsistent with a previous report of downregulated occludin in normal and asthmatic primary human bronchial cells, human lung epithelial cells, and sinonasal epithelial biopsies from patients with chronic rhinosinusitis, induced by the activity of IL-4/13 and histone deacetylases [5, 17, 18, 20, 41, 46]. The functional role of the integral membrane protein occludin is in linking to ZO-1 and the cytoskeleton in order to maintain tight junction integrity [47]. Thus, downregulation of occludin would certainly result in TJ disassembly. In addition, our study further demonstrated that pre-treatment with BE could rescue the decreased expression of occludin in response to IL-4/13 exposure. Besides occludin, the 220-kDa scaffold protein ZO-1 is crucial for linking between occludin and perijunctional actin at the cytoplasmic surface of intercellular junctions [48, 49]. Based on the structural organization of proteins, annular contraction in perijunctional actin could therefore dynamically affect the permeability of the intercellular space. Our results show that IL-4/13-treated cells both pre-incubated with BE and not had no alteration in ZO-1 protein expression, which is not concordant with previous observations [5, 18, 21, 41, 43, 46], but in agreement with those of Saatian *et al*. (2013) [20] (Fig 4). Previous studies reported that IL-4/13 diminished the epithelial barrier function due to decreased ZO-1 expression. These data were obtained from an airway epithelial cell line, lung adenocarcinoma cell line, and primary sinonasal epithelial cell lines. On the other hand, Saatian and colleagues (2013) [20] performed the experiment in 16HBE14o-human bronchial epithelial cells, which was similar to our cell culture system. The inconsistent data may therefore arise from the use of different types of cell line. Furthermore, we noted that BE did not cause any significant changes in ZO-1 protein expression level.

As the beneficial action of BE against TJ disruption induced by IL-4/13 is most likely to occur without changes in occludin and ZO-1 protein expression, it was conceivable that BE might have an influence on the localization or distribution of TJ-associated proteins. We demonstrated that the IL-4/13-treated epithelial cell monolayer had more occludin and ZO-1 protein appearance into cytoplasmic sites rather than co-existing at the paracellular areas in a similar manner to intact cells (Fig 5). Intriguingly, BE prevented IL-4/13-stimulated internalization or mislocalization of these two proteins. The dynamics of TJ protein-mediated breakdown of the epithelial barrier in inflammatory conditions have been previously described [49–52]. Most investigations have focused on intestinal epithelial junctions in animal models of inflammation and tissue biopsies from human patients with different mucosal diseases [50, 53–58]. Less attention has been paid to the role of cytokines in the mobilization of TJ proteins in airway epithelial cells. One study by Saatian and coworkers reported that IL-4/13 disrupts the 16HBE14o- bronchial epithelial barrier by altering the normal structure of the tight junction with unchanged junction protein expression, which was in agreement with our observations.

They speculated that the mechanism is likely involved in cytoskeletal rearrangement, which links to the TJ scaffold proteins such as ZO-1, and thus altered TJ complex assembly.

Having elaborated the mechanism by which BE prevents the impairment of TJ integrity, it is of interest to determine the downstream signaling pathways of Th2 cytokines. It is established that IL-4 and IL-13 exert signals through the shared type 2 IL-4R, and that the JAK/STAT6 pathway is of utmost importance for the classical of its actions in allergic inflammation [13, 22, 25, 40]. We clearly demonstrated that the protective effect of BE against IL-4/13-induced TJ impairment is mediated by interruption of the STAT6-dependent pathway (Fig 6). Interleukin-4/13 induced the upregulation of pSTAT6 expression in vehicle-pretreated 16HBE14o- cell, indicating the activation downstream of the JAK/STAT6 pathway, in which the tyrosine residue of STAT6 is phosphorylated by Janus kinases enzyme to become the phosphorylated form, pSTAT6 [59–61]. Theoretically, activated STAT6 then translocates to target gene promoters in the cell nucleus and thus regulate target gene expression [61, 62]. Our result is consistent with earlier research reporting that STAT6 is required for mediating responses to IL-4 and IL-13 [50, 63–66]. Of interest, it was apparent that BE dose-dependently negated IL-4/13 action, thus reverting pSTAT6 to control levels. In other words, pre-exposure to BE did not alter TJ disruption, as indicated by the greater TEER and lower FITC-dextran permeability in the 20 μM BE + 10 ng/mL each IL-4/13 group vs. the 10 ng/mL each IL-4/13 alone group (Figs 1 and 2).

**Fig 8:**
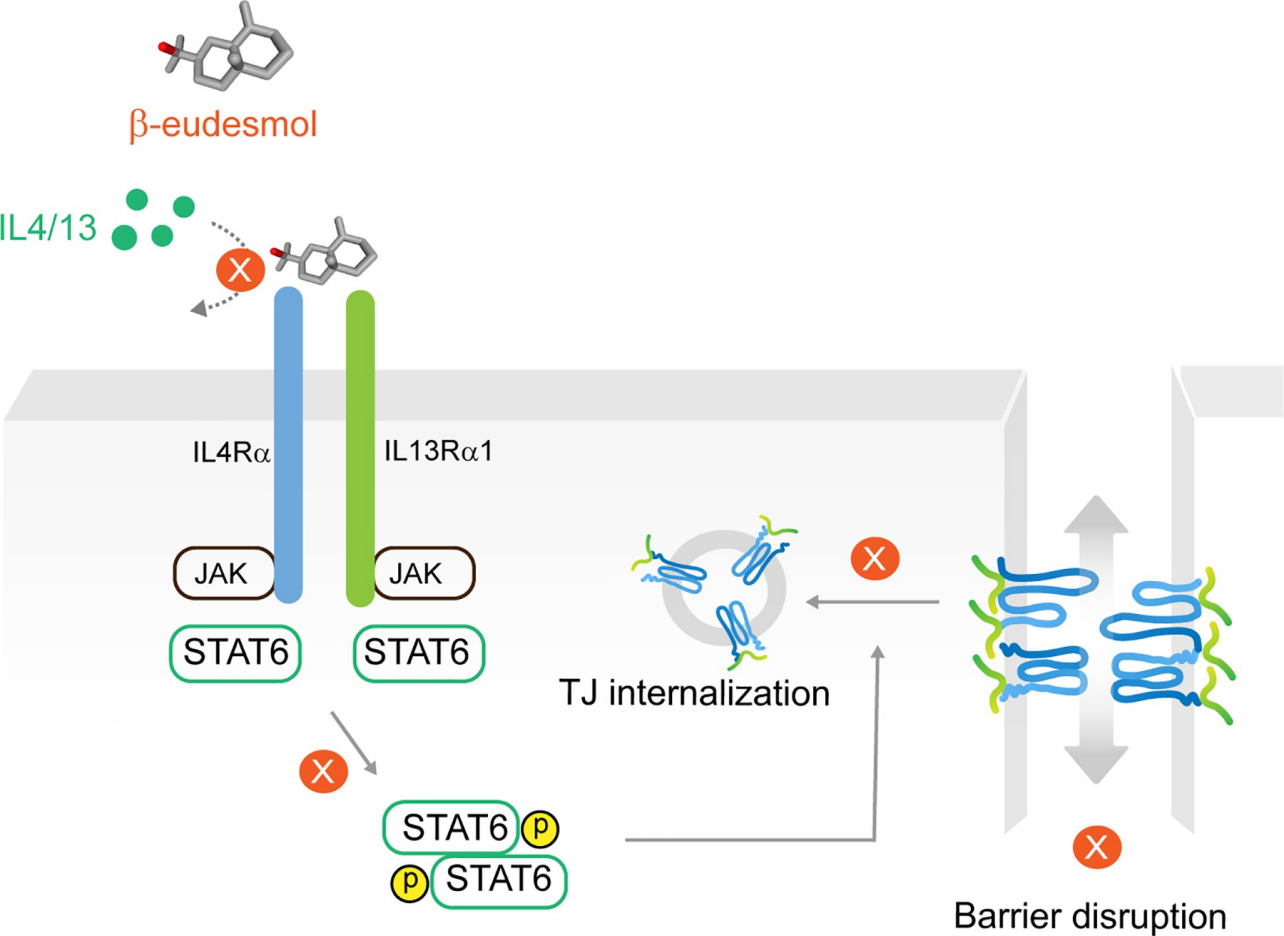
Proposed underpinning mechanism of BE counteracts IL-4/13 signaling to maintain TJ integrity. BE selectively blocks type 2 IL-4R at the site I binding pocket, which can interrupt cytokine actions. This inhibition leads to reduction of STAT6, which induce TJ internalization. Of note, STAT6 possibly regulates TJ proteins through alteration of the cytoskeletal architecture, filamentous actin (F-actin) which contributes to internalization of occludin, and ZO-1. The inhibitory effects of BE (cross sign symbol) on IL-4/13 signaling would therefore suppress TJ disruption.

STAT6-dependent signaling has been proposed to play a crucial role in the differentiation of Th2 cells and immunoglobulin isotype conversion; promotion of proliferation and maturation of B cells; mediation of the expression of MHC-II and IgE as well as activation of mast cells in allergic inflammation [61, 63, 64, 67]. However, there is also evidence that non-hematopoietic STAT6 is associated with epithelial tight junction dysfunction by upregulation of myosin light chain kinase (MLCK) [50]. In the enterocyte, under inflammatory condition, MLCK activation has been implicated in regulation of the peri-junctional actin contraction, resulting in scaffolded protein dynamic and TJ disassembly [51, 68, 69]. Evidence of STAT6-MLCK involvement has thus helped support the involvement of STAT6 in mislocalization of TJ proteins. Building on our results, it is likely that BE prevents IL-4/13-activated STAT6 signaling and internalization of occludin and ZO-1, which may be mediated by MLCK-dependent cytoskeletal remodeling (Fig 8). However, interpreting our findings here is best done in the context of the limitations. First, we proposed the mechanism of BE to be competitive inhibition of IL-4/13 signaling through the JAK/STAT6 pathway via type 2 IL-4R. An alternative mechanism, such as IL-4Rα-dependent PI3K/AKT signaling and IL-13-mediated ERK1/2 MAPK pathway, has not been elucidated. These questions require further investigation. Second, the specific upstream of STAT6 has not yet determined. Since STAT6 can be phosphorylated by either JAK1, JAK2, JAK3 or TYK2, additional experiments are warranted to identify for the certain involvement of JAKs, regarding the BE effect.

The counteraction of IL-4/13 by BE signaling led us to further investigate the protein-ligand interaction. Our *in silico* analysis showed the proposed interacting pocket for BE to be near the site I type 2 IL4R, which has been postulated to be a helical scaffold binding surface on IL-4Rα to bind both IL-4 and IL-13. However, the actual binding sites of BE require confirmation by further physicochemical testing.

From a therapeutic perspective, our study provides the scientific evidence to support the therapeutic application of Orient traditional herb containing BE for allergic airway diseases involving Th2 cytokine-induced epithelial barrier disruption, such as allergic rhinitis and asthma. Further studies on the effect of BE in different disease models driven by type 2 cytokines such as inflammatory bowel diseases, ulcerative colitis, Crohn’s disease, and atopic dermatitis may be indicated.

## Conclusion

We have provided corroborative evidence to establish the anti-inflammatory effect of BE against allergic cytokine-induced barrier disruption in the human airway epithelium. The mechanism of BE is via an interference of type 2 IL-4R, leading to inhibition of the STAT6-dependent signaling pathway and preventing mislocalization of occludin and ZO-1. Further study on BE may provide a naturally-derived drug candidate and provide the rationale for developing novel therapy for allergic inflammatory diseases.

## Supporting information

S1 File Unadjusted images underlying all western blot results reported. (PDF)
