# Supplementary material for: The barrier-protective effect of β-eudesmol against type 2-inflammatory cytokine-induced tight junction disassembly in airway epithelial cells
Source: PLoS One. 2024 Apr 30;19(4):e0302851. doi: 10.1371/journal.pone.0302851 (PMC11060601; doi:10.1371/journal.pone.0302851)

Western blot

The barrier-protective effect of β-eudesmol against type 2-inflammatory cytokines-induced tight junction disassembly in airway epithelia

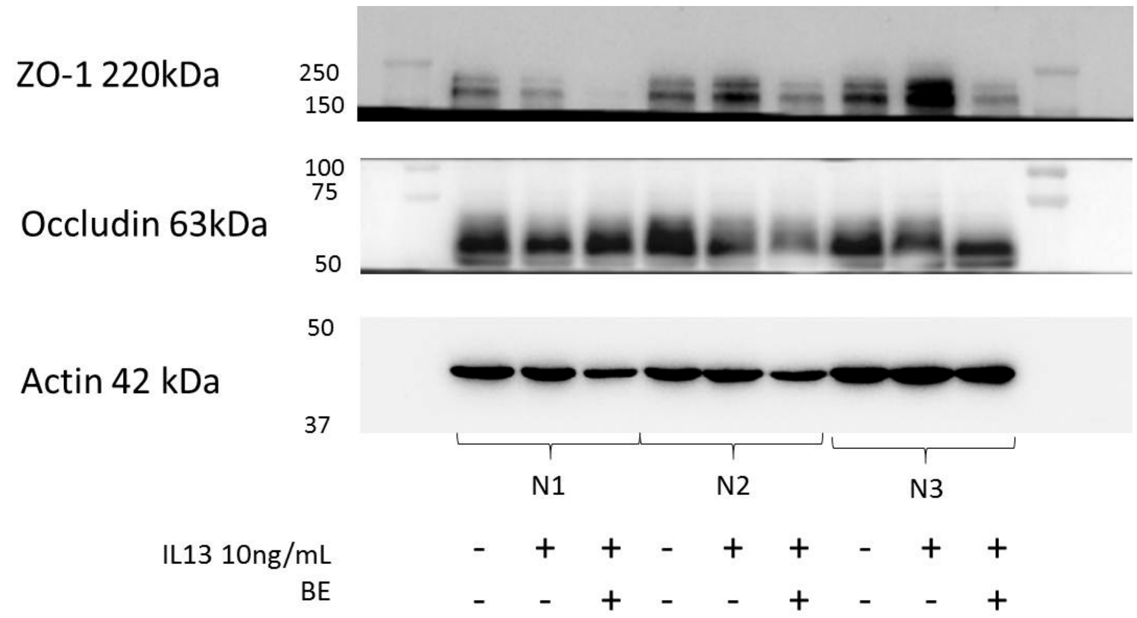

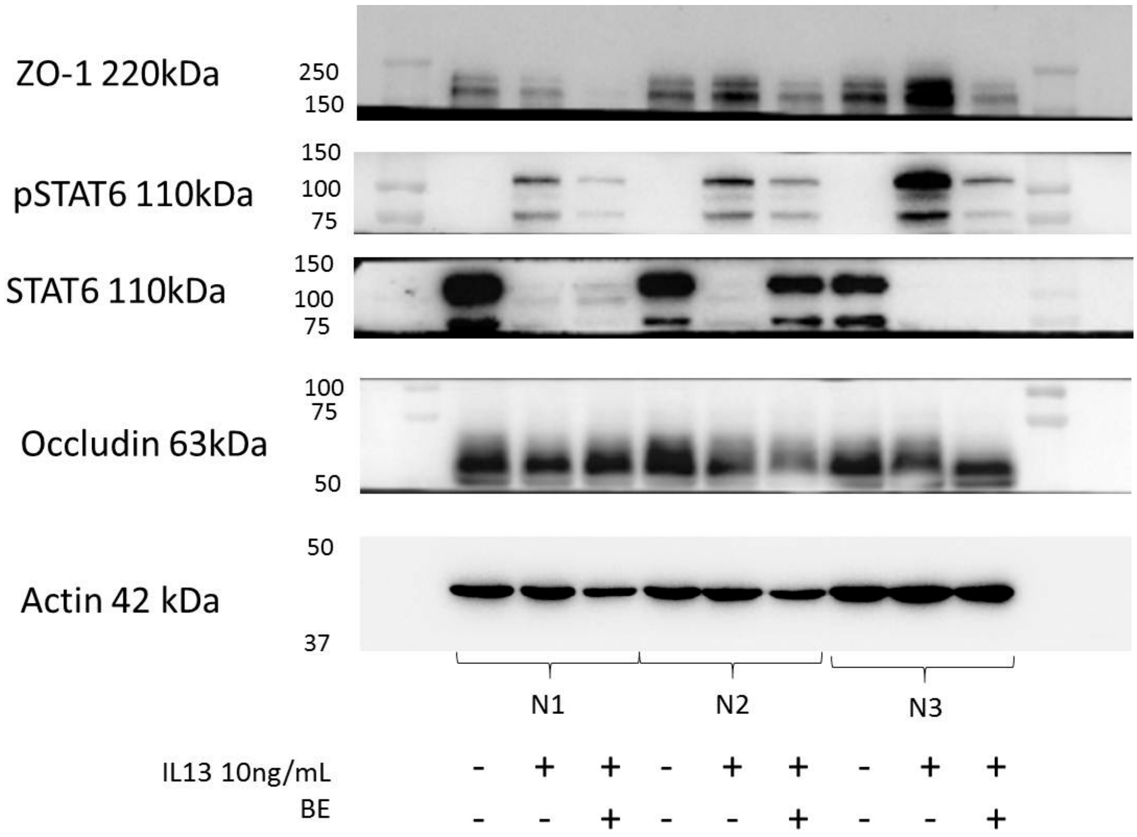

Supplement: S1 File — (PDF) [file pone.0302851.s001.pdf]
